# Primary Synovial Sarcoma of the Prostate: A Case Report and Review of the Literature

**DOI:** 10.7759/cureus.115340

**Published:** 2026-08-28

**Authors:** Abdullah S Alghamdi, Omar M Shaikhomar, Mohammed A Almalki, Mohammed A Basuhail, Salahadin H Lamy

**Affiliations:** 1 Department of Urology, King Fahad Armed Forces Hospital, Jeddah, SAU; 2 Department of Urology, King Abdulaziz Medical City, Jeddah, SAU

**Keywords:** genitourinary oncology, prostatic neoplasm, soft-tissue sarcoma, synovial sarcoma, synovial sarcoma of the prostate

## Abstract

Primary synovial sarcoma of the prostate is exceptionally rare. Its nonspecific presentation and often normal or low prostate-specific antigen (PSA) levels may delay diagnosis. A 49-year-old man presented with lower urinary tract symptoms and gross hematuria. Digital rectal examination revealed a markedly enlarged, firm, irregular prostate. The serum PSA level was 0.34 ng/mL. Pelvic magnetic resonance imaging showed a 9.4 × 7.4 × 6.5 cm heterogeneous prostatic mass with loss of the rectoprostatic fat plane, without definitive evidence of metastatic disease on staging. Transrectal ultrasound-guided biopsy demonstrated a monophasic spindle-cell neoplasm with immunohistochemical findings consistent with synovial sarcoma. Robot-assisted radical prostatectomy was converted to an open approach to facilitate resection. Tumor was identified in tissue from the rectoprostatic margin, while the overall margin status was indeterminate because of tumor fragmentation, raising concern for microscopic residual disease. The patient received adjuvant pelvic radiotherapy followed by doxorubicin and ifosfamide chemotherapy. At 20 months, he remained alive without radiologic evidence of recurrence or metastasis.

Primary prostatic synovial sarcoma should be considered in younger to middle-aged patients with obstructive urinary symptoms, a large prostatic mass, and a normal or low PSA level. Diagnosis relies on histopathologic assessment, supported by immunohistochemistry and molecular testing whenever available. Management should be individualized through multidisciplinary discussion.

## Introduction

Primary synovial sarcoma of the prostate is a rare soft-tissue malignancy, with only a limited number of cases reported in the literature [1]. Although synovial sarcoma most commonly arises in the extremities, it can rarely occur at unusual anatomic sites, including the prostate [2,3]. Its rarity creates important diagnostic and therapeutic challenges, as the clinical presentation often overlaps with more common prostatic conditions such as benign prostatic hyperplasia, prostatitis, or prostatic carcinoma [4].

Patients with prostatic synovial sarcoma may present with nonspecific lower urinary tract symptoms, urinary retention, or hematuria [2,4-6]. Serum prostate-specific antigen (PSA) levels are often normal or not markedly elevated [1]. However, PSA alone is insufficient to establish or exclude the diagnosis. Diagnosis relies on histopathologic assessment, supported by immunohistochemistry, with molecular testing for an SS18 rearrangement or SS18::SSX fusion providing strong diagnostic confirmation [1,4].

Only a small number of primary prostatic synovial sarcoma cases have been documented in the English literature. Compared with synovial sarcoma arising in more typical extremity locations, prostatic synovial sarcoma may pose distinct management challenges because of its deep pelvic location, nonspecific presentation, and potential for delayed diagnosis [7]. Because available data are largely limited to isolated case reports and small literature reviews, further reporting of well-documented cases is important to improve recognition, diagnostic accuracy, and understanding of clinical outcomes.

We report a case of primary synovial sarcoma of the prostate and review the relevant literature, focusing on clinical presentation, diagnostic workup, histopathologic and molecular findings, management, and outcomes.

## Case presentation

A 49-year-old male with no significant medical history presented with a one-week history of worsening lower urinary tract symptoms, including urinary frequency, nocturia, weak urinary stream, and gross hematuria. He denied weight loss, bone pain, bowel symptoms, or other systemic complaints. Digital rectal examination (DRE) revealed a markedly enlarged, firm, and irregular prostate with reduced mobility. Laboratory evaluation demonstrated a PSA level of 0.34 ng/mL, hemoglobin level of 13.9 g/dL, and creatinine level of 78 µmol/L.

**Figure 1:**
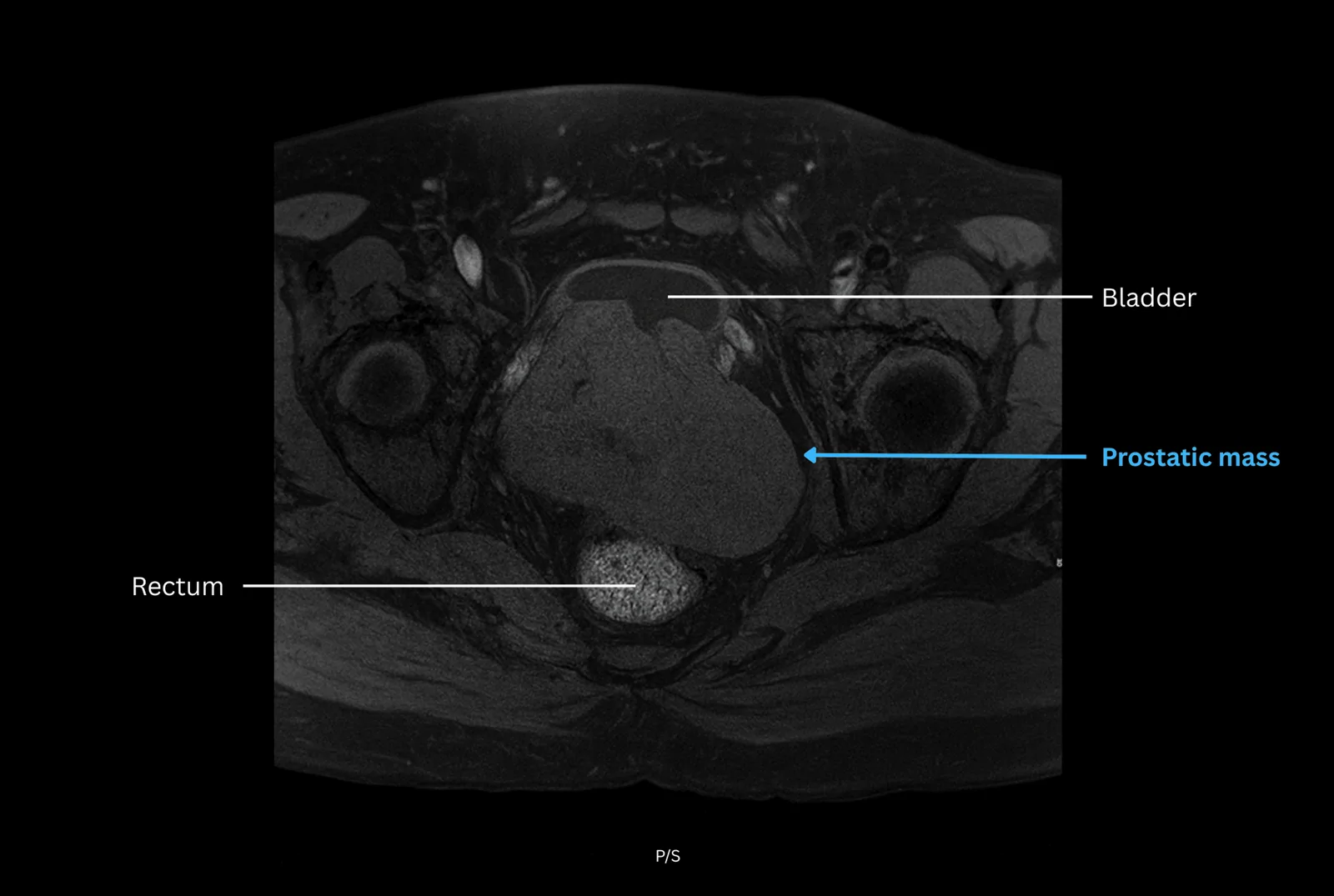
Pelvic MRI showing the prostatic mass and adjacent anatomical structures. The blue arrow indicates the mass, which occupies most of the prostate and nearly obliterates the rectoprostatic fat plane.

Pelvic magnetic resonance imaging (MRI) (Figure 1) demonstrated a large heterogeneous mass measuring 9.4 × 7.4 × 6.5 cm that occupied most of the prostate, contained internal areas of necrosis, and nearly completely obliterated the rectoprostatic fat plane. Positron emission tomography/computed tomography (PET/CT) demonstrated mild fluorodeoxyglucose (FDG) uptake in the external iliac lymph nodes and nonspecific uptake in the right femur. Bone scintigraphy showed no evidence of osseous metastasis. These findings did not provide definitive evidence of nodal or distant metastatic disease. 

Following MRI and PET/CT, transrectal ultrasound (TRUS) was used to guide a prostate biopsy. Histopathological examination showed a neoplastic proliferation composed of monomorphic spindle cells arranged in intersecting fascicles and solid sheets. The cells exhibited a high nuclear-to-cytoplasmic ratio, oval-to-elongated nuclei, and inconspicuous nucleoli. Less cellular areas contained wiry collagen and dystrophic calcifications. 

Immunohistochemical staining showed positivity for CD99, patchy positivity for broad-spectrum cytokeratin AE1/AE3 and epithelial membrane antigen (EMA), and focal positivity for cytokeratin 7 (CK7) and low-molecular-weight cytokeratin (LMWCK). The tumor cells were negative for S100 protein, desmin, BCL-2, STAT6, CD34, broad-spectrum cytokeratin MNF116, and NKX2.2. The histomorphologic and immunohistochemical findings supported the diagnosis of monophasic synovial sarcoma of the prostate. Molecular testing for an SS18 rearrangement or SS18::SSX fusion was not performed because of limited local availability.

**Figure 2:**
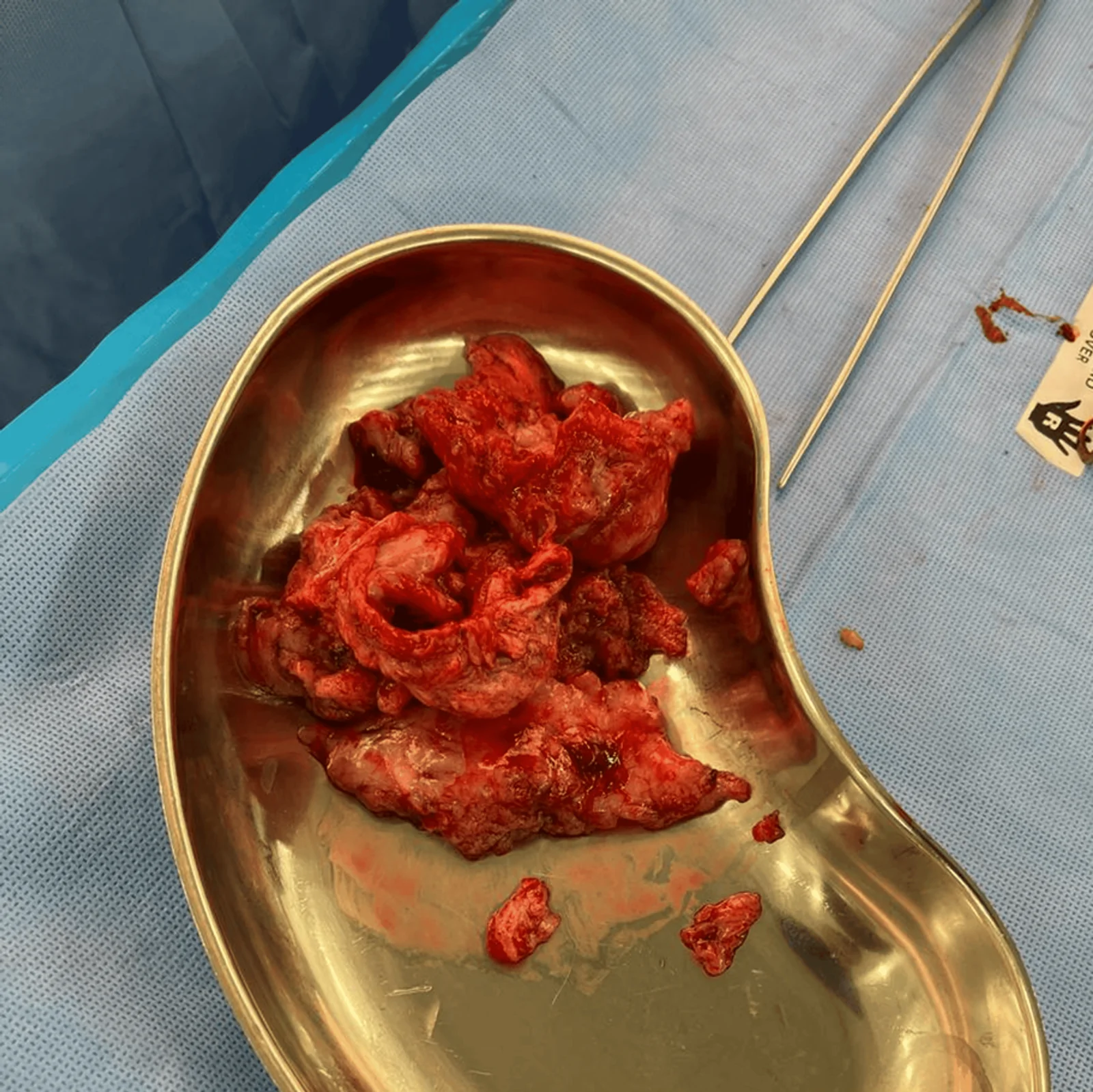
Gross appearance of the fragmented tumor specimen. The resected specimen consisted of multiple fragmented, unoriented tumor pieces. The fragmented appearance resulted from the technically difficult intraoperative dissection.

Considering the absence of definitive metastatic disease on staging investigations, the patient underwent robot-assisted radical prostatectomy. Intraoperatively, the tumor was found to surround the posterior bladder neck. The procedure was therefore converted to an open approach to facilitate tumor resection. During posterior dissection, the tumor appeared to involve the anterior rectal wall and was adherent to adjacent structures. This made the dissection technically difficult and resulted in tumor fragmentation, accounting for the disrupted appearance of the collected specimen shown in Figure 2. 

Dissection of the tumor from the rectum resulted in an approximately 7-cm transmural laceration of the anterior wall of the mid-lower rectum, with its lower edge located around 6-7 cm from the anal verge. A two-layer primary repair of the rectal injury was performed, using a transperitoneal approach followed by a transanal approach, after which a diverting loop ileostomy was created. 

Bilateral external iliac lymph node dissection was performed. Six right and seven left external iliac lymph nodes were dissected, all of which were negative for malignancy (0/13). The excised anterior prostatic fascia and bladder neck specimens were also negative for malignancy. Tissue from the margin between the prostate and rectum was positive for malignancy. However, the overall surgical margin status could not be reliably determined because of tumor fragmentation.

Postoperatively, the patient’s hemoglobin decreased to a nadir of 9.2 g/dL, and he received a transfusion of one unit of packed red blood cells. He otherwise recovered without major postoperative complications. The patient completed pelvic radiotherapy at a total dose of 64 Gy in 32 fractions, followed by combination chemotherapy with doxorubicin and ifosfamide. He tolerated treatment without major complications. 

Surveillance CT of the chest, abdomen, and pelvis was performed approximately every four months. After 20 months of postoperative follow-up, from January 2024 to September 2025, the patient remained alive with no evidence of local recurrence or distant metastasis.

## Discussion

Primary synovial sarcoma of the prostate is exceptionally rare, and the available evidence remains limited to individual case reports and small literature reviews. The cases included in the present review demonstrate that this tumor most often affects younger and middle-aged adults rather than the older population typically affected by prostatic adenocarcinoma [8,9]. Across the 18 cases summarized in Table 1, including the present patient, the median age was approximately 43 years, with a range of 22-63 years.

The clinical presentation is generally nonspecific. Most reported patients presented with lower urinary tract symptoms, dysuria, or urinary retention, whereas hematuria and symptoms related to compression of adjacent pelvic structures were less common. Serum PSA was usually low or only modestly elevated. Among the cases with reported PSA values, the median PSA level was 1.03 ng/mL. The PSA level of 0.34 ng/mL in the present case was consistent with the reported pattern. A low PSA level in a patient with a large prostatic mass should raise consideration of a nonepithelial prostatic neoplasm, although it cannot distinguish synovial sarcoma from other mesenchymal tumors.

Large tumor size and local extension were common among the reported cases. The median maximum tumor dimension was 8.5 cm, and several tumors involved or compressed adjacent pelvic structures, including the bladder, rectum, seminal vesicles, and surrounding soft tissues. The present tumor measured 9.4 cm in greatest dimension and showed loss of the rectoprostatic fat plane on MRI, corresponding intraoperatively to adherence to and involvement of the anterior rectal wall. These findings highlight the importance of careful preoperative assessment and multidisciplinary planning when involvement of adjacent pelvic organs is suspected. The clinical and radiologic findings of the reported cases are summarized in Table 1. 

**Table 1:** Clinical presentation and imaging findings in reported cases of primary prostatic synovial sarcoma and the present case. Tumor dimension denotes the largest reported dimension. The source of measurement, whether imaging or pathologic examination, was not consistently specified in the original publications. CT, computed tomography; FDG, fluorodeoxyglucose; MRI, magnetic resonance imaging; NR, not reported; PET, positron emission tomography; PSA, prostate-specific antigen; TRUS, transrectal ultrasound; US, ultrasonography.

Case no.	Author, Year	Age (years)	Clinical presentation	Serum PSA (ng/mL)	Tumor dimension (cm)	Tumor extent and key imaging findings
1	Iwasaki et al., 1999 [2]	37	Gross hematuria; dysuria	NR	11	CT: large pelvic mass compressing bladder and rectum. MRI: prostatic mass extending into retrovesical soft tissue.
2	Fritsch et al., 2000 [10]	34	Prostatism	NR	7	Initial imaging and disease extent NR.
3	Shirakawa et al., 2002 [11]	52	Urinary retention	0.9	7	Pelvic CT/MRI: mass arising from right prostatic fascia.
4	Williams et al., 2004 [12]	63	Lower urinary tract symptoms	0.5	8.5	CT/MRI: mass extending from the mid-prostate to the penile base, with corpora cavernosa invasion. No pelvic lymphadenopathy or distant metastases.
5	Pan and Chang, 2006 [6]	44	Lower urinary tract symptoms	2.91	6	Pelvic CT: well-defined heterogeneous mass involving right side of prostate.
6	Jun et al., 2008 (case 1) [13]	46	Dysuria	0.35	6	CT: posterior prostatic mass extending into pelvic soft tissue. No distant metastasis.
7	Jun et al., 2008 (case 2) [13]	44	Progressive dysuria	1.19	12	US: large prostatic mass extending into pelvic soft tissue.
8	Dhabalia et al., 2009 [8]	25	Acute urinary retention; blood-stained rectal discharge	1.7	9.5	MRI: solid prostatic tumor involving rectum. No significant lymphadenopathy or distant metastasis. Bone scan: negative.
9	Zhang et al., 2014 [9]	22	Urinary retention; dysuria; urinary frequency; nocturia	1.20	14	Pelvic CT/MRI: prostatic fascial mass and a separate right groin mass. CT/chest radiography: pulmonary and hepatic metastases.
10	Olivetti et al., 2015 [14]	46	Urinary retention	1.03	8.5	CT/MRI: well-circumscribed mass of prostatic origin with cystic, solid, and myxoid components, extending into the retrovesical soft tissues and compressing the right seminal vesicle. No metastases.
11	Maleki et al., 2017 [15]	38	Back pain radiating to abdomen; constipation; lower abdominal pain	0.58	9.5	Pelvic CT: complex hypodense periprostatic mass posterior to bladder, with involvement of rectum, seminal vesicles, and prostate. No significant pelvic or retroperitoneal lymphadenopathy.
12	Ehsanullah et al., 2022 [16]	52	Urinary retention	NR	9.6	CT: enlarged prostate with mass effect on rectum and bladder. MRI: heterogeneous partially necrotic left prostatic mass invading rectal wall. PET: FDG-avid tumor with suspicious left iliac nodal metastases.
13	Hou et al., 2022 [1]	42	Dysuria; interrupted urinary stream; nocturia	0.81	6.7	MRI: irregular right posterior prostatic mass with rectal involvement and right seminal vesicle invasion causing urethral compression/displacement.
14	Bandegudda et al., 2022 [4]	35	Urinary retention	NR	4.9	TRUS: diffusely enlarged prostate with altered echotexture and left lateralization. MRI: diffusion-restricting lesion with heterogeneous enhancement and minimal extraprostatic extension to bladder base.
15	Emadi Torghabeh et al., 2022 [5]	30	Urinary obstructive symptoms; progressive suprapubic pain	5.94	5.7	MRI: heterogeneous prostatic fascial mass between rectum and bladder with bladder floor extension, lymphadenopathy anterior to the sacrum, and necrotic areas. CT/bone scan: multiple lung metastases and left iliac bone metastasis.
16	Shi et al., 2023 [3]	58	Asymptomatic	NR (reported as normal)	4.9	US: left-sided mixed-echo prostatic mass compressing left seminal vesicle. MRI: left central-zone prostatic mass protruding posteriorly/leftward with diffusion restriction. CT: no regional lymph node or distant metastasis.
17	Wang et al., 2025 [7]	36	Dysuria; urinary hesitancy; urinary retention; nocturia	6.33	10.3	TRUS: irregular prostatic mass extending into the bladder. CT: enlarged heterogeneous prostate with invasion of adjacent pelvic structures. MRI: prostatic/bladder wall masses with rectal mesenteric invasion and pelvic floor compression. PET: hypermetabolic prostatic mass with multiple pulmonary nodules and other body site lesions.
18	Present case	49	Gross hematuria; urinary frequency; nocturia; weak urinary stream	0.34	9.4	MRI: heterogeneous prostatic mass with necrosis and loss of rectoprostatic fat plane. PET: mild uptake in external iliac lymph nodes and nonspecific right femoral uptake. No definite distant metastasis. Bone scan: negative.

Diagnosis is particularly challenging in monophasic tumors because their spindle-cell morphology overlaps with several other primary and secondary prostatic neoplasms. Relevant differential diagnoses include prostatic stromal sarcoma, malignant peripheral nerve sheath tumor, and other spindle-cell malignancies. In the present case, the monomorphic fascicular spindle-cell morphology, together with CD99 expression and patchy or focal expression of epithelial markers, supported monophasic synovial sarcoma. Nevertheless, these histomorphologic and immunohistochemical features are supportive rather than individually diagnostic.

Molecular testing for an SS18 rearrangement or SS18::SSX fusion provides strong support for the diagnosis. Molecular testing was reported in 13 of the 18 cases included in the current review. Molecular testing was not performed in the present patient because of limited local availability. The diagnosis was therefore based on the histomorphologic and immunohistochemical findings. The absence of molecular testing represents a limitation of the diagnostic evaluation. The histopathologic, immunohistochemical, and molecular findings of the reported cases are summarized in Table 2.

**Table 2:** Histopathologic and molecular findings, treatment, and outcomes in reported cases of primary prostatic synovial sarcoma and the present case. Older publications used the terms SYT and SYT-SSX, the corresponding current nomenclature is SS18 and SS18::SSX, respectively. Molecular findings are presented according to the original reports, with current nomenclature added in parentheses where appropriate. Fusion-specific SS18-SSX immunohistochemical staining is reported in the immunohistochemistry column and is not classified as molecular testing. AE1/AE3, cytokeratin antibody cocktail; AFP, alpha-fetoprotein; AR, androgen receptor; BCL-2, B-cell lymphoma 2; CAM5.2, low-molecular-weight cytokeratin antibody; CD31, CD34, CD56, CD99, and CD117, cluster of differentiation 31, 34, 56, 99, and 117, respectively; CK, cytokeratin; CK7, CK18, CK19, and CK20, cytokeratins 7, 18, 19, and 20, respectively; EMA, epithelial membrane antigen; FISH, fluorescence in situ hybridization; FLI1, Friend leukemia integration 1 transcription factor; HHF35, muscle-specific actin antibody; INI1, integrase interactor 1 (SMARCB1); Ki-67, proliferation marker; LMWCK, low-molecular-weight cytokeratin; MIC2, historical designation for CD99; MNF116, broad-spectrum cytokeratin antibody; MyoD1, myogenic differentiation 1; NKX2.2, NK2 homeobox 2; NR, not reported; NSE, neuron-specific enolase; P504S, alpha-methylacyl-CoA racemase; PR, progesterone receptor; PSA, prostate-specific antigen; RT-PCR, reverse transcription polymerase chain reaction; S100, S100 protein; SMA, smooth muscle actin; SOX10, SRY-box transcription factor 10; SS18, SS18 subunit of the BAF chromatin-remodeling complex; SS18-SSX, SS18-SSX fusion protein; SSX, synovial sarcoma X breakpoint family; STAT6, signal transducer and activator of transcription 6; SYT, historical designation for SS18; TLE1, transducin-like enhancer of split 1; WT1, Wilms tumor 1; α-SMA, alpha-smooth muscle actin.

Case no.	Author, Year	Histologic subtype	Immunohistochemistry	Molecular testing	Treatment	Resection, margin, and nodal status	Follow-up duration	Outcome
1	Iwasaki et al., 1999 [2]	Monophasic synovial sarcoma	Vimentin+, EMA (focal)+; keratin−, muscle-specific actin−, α-SMA−, desmin−, S100−, NSE−, CD34−	t(X;18)(p11.2;q11.2) detected by cytogenetic analysis and FISH	Neoadjuvant vincristine/cisplatin/actinomycin D/etoposide chemotherapy; radical prostatocystectomy; re-excision for local recurrence	Negative surgical margins	32 months	Died of disease
2	Fritsch et al., 2000 [10]	Poorly differentiated synovial sarcoma	Vimentin (diffuse)+, EMA (focal)+; muscle-specific actin−, desmin−, MIC2−, CAM5.2−, AE1/AE3−	SYT-SSX (SS18::SSX) fusion transcript detected by RT-PCR	Radical cystoprostatectomy with en bloc rectal resection; doxorubicin/cisplatin chemotherapy for presacral recurrence; pulmonary wedge resection followed by fluorouracil/etoposide chemotherapy	Negative surgical margins; bladder, rectum, and pelvic lymph nodes negative	35 months	Died of disease
3	Shirakawa et al., 2002 [11]	Monophasic synovial sarcoma	Vimentin+, EMA (focal)+; α-SMA−, desmin−, S100−	t(X;18)(p11.2;q11.2) detected by FISH	Radical retropubic prostatectomy; adjuvant doxorubicin/ifosfamide chemotherapy	Negative surgical margins	6 months	No evidence of disease
4	Williams et al., 2004 [12]	Monophasic synovial sarcoma	Vimentin+, AE1/AE3+, CAM5.2+, CK7+, BCL-2+, calponin+, S100+; EMA−, CD99−, MIC2−, SMA−, desmin−, CD34−, CD117−	SYT-SSX1 (SS18::SSX1) fusion variant associated with t(X;18) detected by cytogenetic analysis	Preoperative external beam radiotherapy; anterior pelvic exenteration with en bloc penectomy, pubectomy, and ileal conduit urinary diversion	Positive right pelvic soft-tissue margin; lymph nodes negative	NR	NR
5	Pan and Chang, 2006 [6]	Monophasic synovial sarcoma	Cytokeratin (focal)+, BCL-2+, CD99 (focal)+; EMA−, SMA−, HHF35−, desmin−, S100−, CD34−	SYT-SSX2 (SS18::SSX2) fusion transcript detected by RT-PCR	Radical prostatectomy	Margin status NR; tumor confined to prostate	NR	NR
6	Jun et al., 2008 (case 1) [13]	Monophasic synovial sarcoma	Vimentin+, CK (focal)+, EMA (focal)+, E-cadherin+, BCL-2+, CD99+; SMA−, desmin−, S100−, CD34−, CD117−, PSA−, calretinin−	SYT-SSX (SS18::SSX) fusion detected by RT-PCR	Radical prostatectomy; adjuvant doxorubicin/ifosfamide chemotherapy	Negative surgical margins	8 months	No evidence of disease
7	Jun et al., 2008 (case 2) [13]	Monophasic synovial sarcoma	Vimentin+, CK (focal)+, E-cadherin+, BCL-2+, CD99+; EMA−, SMA−, desmin−, S100−, CD34−, CD117−, PSA−, calretinin−	SYT-SSX (SS18::SSX) fusion detected by RT-PCR	Surgical resection	Incomplete resection due to bladder, rectal, and pelvic soft-tissue invasion	11 months	Died of disease
8	Dhabalia et al., 2009 [8]	Synovial sarcoma; subtype NR	Vimentin+, cytokeratin (focal)+, BCL-2+; calponin−, S100−	NR	Total pelvic exenteration with end sigmoid colostomy, and ileal conduit urinary diversion	Negative surgical margins	18 months	No evidence of disease
9	Zhang et al., 2014 [9]	Synovial sarcoma; subtype NR	Vimentin+, CD99+; α-SMA−, desmin−, S100−	SYT-SSX (SS18::SSX) fusion transcript detected by RT-PCR	No treatment; patient declined surgery, chemotherapy, and radiotherapy	NA	3 months	Died of disease
10	Olivetti et al., 2015 [14]	Monophasic synovial sarcoma	Pan-cytokeratin (focal)+, BCL-2+, CD99+, CD56+; SMA−, desmin−, S100−, CD34−	SYT (SS18) rearrangement associated with t(X;18)(p11;q11) detected by FISH	Debulking surgery; postoperative epirubicin/ifosfamide chemotherapy	Positive surgical margins; incomplete resection	3 months	Alive with disease
11	Maleki et al., 2017 [15]	Monophasic synovial sarcoma	Vimentin+, AE1/AE3+, CAM5.2+, CK7 (focal)+, CK19 (focal)+, EMA (focal)+, BCL-2+, CD99+, calretinin (focal)+; CK20−, SMA−, desmin−, S100−, CD31−, CD34−, WT1−, chromogranin−, synaptophysin−, AFP−	SYT (SS18) rearrangement detected by break-apart FISH	Robot-assisted laparoscopic radical prostatectomy; concomitant chemoradiotherapy	Margin status NR; pelvic lymph nodes negative	24 months	Died of disease
12	Ehsanullah et al., 2022 [16]	Synovial sarcoma; subtype NR	SS18-SSX+, TLE1+; pan-cytokeratin−, AE1/AE3−, desmin−, SOX10−, CD34−, CD117−, STAT6−, PR−	NR	NR	NR	NR	NR
13	Hou et al., 2022 [1]	Monophasic synovial sarcoma	SS18-SSX+, SSX+, vimentin+, BCL-2+, CD99+; CK−, CAM5.2−, CK7−, CK19−, EMA−, SMA−, desmin−, MyoD1−, myogenin−, S100−, CD34−, CD117−, calretinin−, WT1−	SS18 rearrangement detected by break-apart FISH	No treatment	NA	18 months	Died of disease
14	Bandegudda et al., 2022 [4]	Synovial sarcoma; subtype NR	SS18-SSX (focal)+, TLE1+, BCL-2+, CD99+, FLI1+; EMA−, CD34−, PR−, synaptophysin−; INI1 retained	NR	Radical cystoprostatectomy with anterior rectal wall resection, bilateral pelvic lymph node dissection, and ileal conduit urinary diversion; adjuvant doxorubicin/ifosfamide chemotherapy	Negative surgical margins; lymph nodes negative	NR	Died of disease
15	Emadi Torghabeh et al., 2022 [5]	High-grade synovial sarcoma	TLE1+, EMA+; S100−, CD34−, PSA−, p63−, CK7−	NR	Palliative doxorubicin/ifosfamide/dacarbazine/mesna chemotherapy	NA	18 months	Died of disease
16	Shi et al., 2023 [3]	Biphasic synovial sarcoma with concurrent acinar adenocarcinoma	Sarcomatous component: TLE1+, CKH+, CK18+, EMA+, BCL-2+; Ki-67 index 46%; desmin−, S100−, CD34−, CD117−. Glandular component: PSA+, P504S+; CKH−, p63−	SYT-SSX (SS18::SSX) fusion detected by FISH	Radical prostatectomy; adjuvant doxorubicin/ ifosfamide/mesna chemotherapy; radiotherapy	Margin status NR	2 months	No evidence of disease
17	Wang et al., 2025 [7]	Synovial sarcoma; subtype NR	TLE1+, EMA+, SMA+, AR+, PR+, INI1+; Ki-67 index 60%; CK−, desmin−, myogenin−, S100−, CD34−	SS18 rearrangement detected by break-apart FISH	Liposomal doxorubicin/ifosfamide chemotherapy	NA	2 months	Alive with disease
18	Present case	Monophasic synovial sarcoma	CD99+; AE1/AE3 (patchy)+, EMA (patchy)+, CK7 (focal)+, LMWCK (focal)+; S100−, desmin−, BCL-2−, STAT6−, CD34−, MNF116−, NKX2.2−	NR	Radical prostatectomy with bilateral pelvic lymph node dissection, rectal repair, and diverting loop ileostomy; adjuvant radiotherapy; doxorubicin/ifosfamide chemotherapy	Indeterminate margin status; rectoprostatic margin tissue positive for malignancy; pelvic lymph nodes negative	20 months	No evidence of disease

Because of the rarity of primary prostatic synovial sarcoma, no standardized management approach has been established. Surgery was the main treatment for localized tumors in the reported cases, although the extent of resection varied from radical prostatectomy to cystoprostatectomy or total pelvic exenteration according to involvement of adjacent organs. In the present case, radical prostatectomy was selected because staging investigations showed no definitive metastatic disease. The marked adherence of the tumor to the rectum complicated resection and resulted in fragmentation of the tumor and rectal injury requiring primary repair and diverting ileostomy. This experience illustrates the technical difficulty of achieving complete resection when a large prostatic sarcoma adheres to adjacent structures.

The roles of chemotherapy and radiotherapy in primary prostatic synovial sarcoma remain uncertain. Doxorubicin- and ifosfamide-based chemotherapy has been used in several reported cases, either as adjuvant therapy or for recurrent, unresectable, or metastatic disease, whereas radiotherapy has been used less consistently. In the present case, postoperative pelvic radiotherapy followed by doxorubicin and ifosfamide was administered because of local involvement of adjacent pelvic structures, tumor identified in tissue from the rectoprostatic margin, and an indeterminate overall margin status due to tumor fragmentation. These findings raised concern for microscopic residual disease along the rectoprostatic plane. At 20 months after surgery, the patient remained alive without radiologic evidence of local recurrence or distant metastasis. The management strategies, follow-up duration, and outcomes of the reported cases are summarized in Table 2.

Interpretation of the reported outcomes is limited by missing data in several reports, heterogeneous treatment approaches, variable diagnostic confirmation, and generally short follow-up periods. Several patients remained free of disease after surgical treatment, whereas others developed recurrent or progressive disease and died of the disease. The small number of cases and substantial clinical heterogeneity hinder reliable comparisons between treatments or identification of independent prognostic factors.

## Conclusions

Primary prostatic synovial sarcoma, although rare, should be considered in younger to middle-aged patients presenting with obstructive urinary symptoms, a large prostatic mass, and a normal or low PSA level. A normal PSA level does not exclude clinically significant prostatic malignancy, and DRE remains an essential component of the initial evaluation. Diagnosis relies on histopathologic assessment, supported by immunohistochemistry and molecular testing whenever available. Management should be individualized through multidisciplinary discussion, with attention to tumor extent, the feasibility of complete resection, and the potential roles of chemotherapy and radiotherapy.
